# Role of morphological changes in newly born granule cells of hippocampus after status epilepticus induced by pilocarpine in hyperexcitability

**DOI:** 10.1186/1471-2202-13-S1-P56

**Published:** 2012-07-16

**Authors:** Julian Tejada, Norberto Garcia-Cairasco, Antonio C Roque

**Affiliations:** 1 Departamento de Física, FFCLRP, Universidade de São Paulo, Ribeirão Preto, SP, 14040-901, Brazil; 2 Departamento de Fisiologia, FMRP, Universidade de São Paulo, Ribeirão Preto, SP, 14049-900, Brazil

## 

**Figure 1:**
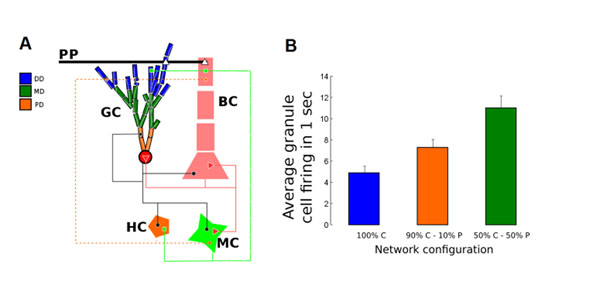
Basic circuit diagram and effect of PILO-treated GC morphological changes on the average GCs response. **A**. Schematic view of the network (adapted from [1]). GC: granule cell; BC: basket cell; HC: HIPP cell; MC: mossy cell. DD: distal dendrites; MD: medial dendrites; PD: proximal dendrites. **B**. Average GC firing in the network configurations with only control GCs (100% C), 10% PILO-treated GCs (90% C – 10% P), and 50% PILO-treated GCs (50% C – 50% P).

Newly born dentate gyrus (DG) granule cells (GCs) after *Status Epilecticus* induced by pilocarpine (PILO) exhibit morphological changes including narrower arborizations, greater number of branches and more endings in the inner molecular layer (IML) [1]. The increased concentration of dendrites in the IML where granule cell axons (mossy fibers) sprout in epileptic animals and make extensive recurrent excitatory synapses may contribute to enhance the DG hyperexcitability. A previous DG network model has shown that mossy fiber sprouting has a crucial role on hyperexcitability with only 10% sprouting being enough to generate spread of activity to all GCs in the network [2]. However, the additional effect of GC morphological changes on DG hyperexcitability is as yet unknown. Here we used the DG model [2] to evaluate the effect of different GC morphologies on the network activity. We replaced all reduced GC models of [2] with morphologically detailed models coming from tridimensional reconstructions of newly born doublecortin-positive DG GCs [1]. The scheme of the network connections is shown in Figure 1A. Our sample of morphologically reconstructed GCs includes 20 from PILO-treated rats and 20 from control rats. Our cell models were constructed in NEURON and their ionic channels and maximum conductance distributions were the same as [2] taking into consideration the shorter arborizations of the PILO-treated cells. Our “control” network model is exactly the same as the topographic ring network of [2] with 10% sprouting available in ModelDB with the 500 original GC models replaced by morphologically reconstructed cells randomly chosen from our control sample. From our control network model we constructed two other models: one with 10% of the GCs (chosen randomly) replaced by cells chosen randomly from our PILO-treated sample and the other with the fraction of PILO-treated GC cells increased to 50%. The networks were submitted to focal perforant-path stimulations as in [2] and to obtain averages we ran 100 simulations for each network (connections between cells were created anew before each run). Our control network model produced a response similar to the one of the original model [2], showing that the insertion of GCs with realistic morphologies did not affect the 10% sprouting DG hyperexcitability (Figure 1 B, 100% C). In contrast, when a few amount of PILO-treated GCs were inserted the excitability of the network increased (Figure 1B , 90% C – 10% P) and the increase was higher when the amount of PILO-treated GCs was larger (Figure 1 B, 50% C – 50% P). However, the effect of the insertion of PILO-treated GCs was only visible in combination with mossy fiber sprouting. Our results suggest that changes in GC morphology alone are not enough to affect the DG hyperexcitability but when these changes occur in the presence of other alterations such as mossy fiber sprouting they could enhance the DG hyperexcitability.
